# Overexpression of Nta-miR6155 confers resistance to *Phytophthora nicotianae* and regulates growth in tobacco (*Nicotiana tabacum* L.)

**DOI:** 10.3389/fpls.2023.1281373

**Published:** 2023-11-20

**Authors:** Kaiyue Yang, Yuanyuan Huang, Zexuan Li, Qian Zeng, Xiumei Dai, Jun Lv, Xuefeng Zong, Kexuan Deng, Jiankui Zhang

**Affiliations:** ^1^ College of Agronomy and Biotechnology, Southwest University, Chongqing, China; ^2^ Engineering Research Center of South Upland Agriculture, Ministry of Education, Chongqing, China; ^3^ Chongqing Tobacco Science Research Institute, Chongqing, China

**Keywords:** *Nicotiana tabacum*, *Phytophthora nicotianae*, tobacco black shank, Nta-miR6155, NtCIPK18, nitrogen

## Abstract

Tobacco black shank induced by *Phytophthora nicotianae* causes significant yield losses in tobacco plants. MicroRNAs (miRNAs) play a pivotal role in plant biotic stress responses and have great potential in tobacco breeding for disease resistance. However, the roles of miRNAs in tobacco plants in response to *P. nicotianae* infection has not been well characterized. In this study, we found that Nta-miR6155, a miRNA specific to *Solanaceae* crops, was significantly induced in *P. nicotianae* infected tobacco. Some of predicted target genes of Nta-miR6155 were also observed to be involved in disease resistance. To further investigate the function of miR6155 in tobacco during *P. nicotianae* infection, Nta-miR6155 overexpression plants (miR6155-OE) were generated in the Honghua Dajinyuan tobacco variety (HD, the main cultivated tobacco variety in China). We found that the Nta-miR6155 overexpression enhanced the resistance in tobacco towards *P. nicotianae* infections. The level of reactive oxygen species (ROS) was significantly lower and antioxidant enzyme activities were significantly higher in miR6155-OE plants than those in control HD plants during *P. nicotianae* infection. In addition, we found that the accumulation of salicylic acid and the expression of salicylic acid biosynthesis and signal transduction-related genes is significantly higher in miR6155-OE plants in comparison to the control HD plants. Furthermore, we found that Nta-miR6155 cleaved target genes *NtCIPK18* to modulate resistance towards *P. nicotianae* in tobacco plants. Additionally, phenotypic analysis of miR6155-OE plants showed that Nta-miR6155 could inhibit the growth of tobacco by suppressing nitrogen uptake and photosynthesis. In conclusion, our findings indicated that miR6155 plays a crucial role in the regulation of growth and resistance against *P. nicotianae* infections in tobacco plants.

## 1 Introduction

Tobacco (*Nicotiana tabacum* L.) is a high value plant of the *Solanaceae* family (Meng et al., 2021). *Phytophthora nicotianae* induced tobacco black shank is one of the most destructive diseases that causes huge economic losses in tobacco production (Liu et al., 2022). *P. nicotianae*, belonging to the phylum Oomycota, is parasitic on plants or resides in the soil in the form of hyphae or chlamydospores (Jin and Shew, 2021). Under favorable growth conditions, the spores of *P. nicotianae* germinate and invade plants through stomata or wounds. After a period of growth, they release a large number of zoospores to infect other tobacco plants. *P. nicotianae* can infect tobacco at different stages of tobacco growth and development, and the most typical infectious symptom is a black stem in tobacco (Yang et al., 2017). The main control measures for tobacco black shank are planting disease-resistant varieties, optimizing cultivation and management measures, and chemical and biological control methods (Guo et al., 2020). Among them, planting disease-resistant varieties is one of the most economical and effective methods. Therefore, it is important to identify *P. nicotianae* resistance genes and breed highly resistant varieties of tobacco.

MicroRNAs (miRNAs) play important roles in regulating plant growth and development and stress response by shearing target messengerRNAs (mRNAs) genes or inhibiting the translation of target mRNAs (Zhu et al., 2020; Han and Wang, 2022). In recent years, many miRNAs have been discovered through miRNA sequencing (Evers et al., 2015), and the biosynthesis, mechanism of action, and functions of plant miRNAs have gradually been revealed (Song et al., 2019; Li and Yu, 2021). Since Lee et al. identified the first miRNA lin-4 in *Caenorhabditis elegans* (Lee et al., 1993), thousands of plant and animal miRNAs have been discovered (Llave et al., 2002; Mette et al., 2002; Reinhart et al., 2002). The process by which plant miRNAs modulate stability of their target mRNAs consists of three main steps: transcription of miRNAs, processing and maturation, and assembly of functional complexes to cleave the target mRNAs (Addo-Quaye et al., 2008; German et al., 2008; Kurihara and Watanabe, 2010; Achkar et al., 2016). However, when the degree of complementarity between the miRNA and target mRNA is low, the miRNA is unable to mediate cleavage and will then inhibit its translation by binding to the target mRNA (Zamore and Haley, 2005).

In recent decades, the function of plant miRNAs in regulating plant biotic stress has been revealed (Zheng et al., 2020; Song et al., 2021). Overexpression of miR394 downregulates the transcription level of its target gene, *LCR* (Leaf Curling Responsiveness), and ultimately decreases the resistance against *Phytophthora infestans* in tomato plants (Zhang et al., 2021). In potatoes, overexpression of miR482e was reported to increase sensitivity to *Verticillium dahliae* infection (Yang et al., 2015). Overexpression of Osa-miR162a enhances resistance towards *Magnaporthe oryzae* and fine-tunes the effect on yield in rice plant (Li et al., 2020). Recently, Yang et al. showed that the overexpression of miR395 could promote resistance to *Xanthomonas oryzae pv. oryzae* (*Xoo*) and *X. oryzae pv. oryzicola* (*Xoc*) by regulating sulfate accumulation and distribution in rice (Yang et al., 2022). MiRNAs have also been found to be involved in biotic stress tobacco response in tobacco plants. The expression profile of miRNA is significantly affected in tobacco following potato virus Y infection (Guo et al., 2017). Overexpression of miR396 has been reported with enhanced tobacco susceptibility to *P. nicotianae* (Chen et al., 2015). However, only a few studies have been conducted on the role of miRNAs in the response of tobacco to *P. nicotianae*.

In view of the above, the present study was designed to reveal the function of miRNA in tobacco in response to *P. nicotianae* infection and provide new genetic resources for breeding of *P. nicotianae* resistant tobacco varieties.

## 2 Methods

### 2.1 Plant materials, *P. nicotianae* strains, and culture conditions

Seeds of *N. benthamiana* and Honghua Dajinyuan (HD, a main cultivated *N. tabacum* (tobacco) variety in China) were sown in sterile soil and grown in a greenhouse at a temperature of 25°C, 16/8 hours dark and 10000 LX light intensity.

*P. nicotianae* strain WL1-7 was cultured on 10% V8 vegetable juice medium (100 mL/L vegetable juice, 0.2 g/L CaCO_3_, 20 g/L agar) at 25°C in a growth chamber without light.

### 2.2 Construction of Nta-miR6155 and *NtCIPK18* overexpression vector

Primers for generation of Nta-miR6155 overexpression vector were designed based on the Nta-miR6155 genome sequence by using Primer Premier 5.0 software, and the adaptor for seamless cloning was added in primers (**Table S1**). Tobacco genomic DNA was extracted using the CTAB method and the precursor gene of Nta-miR6155 was cloned using tobacco genomic DNA as a template. The modified pCAMBIA2300 vector was digested by XbaI and AscI restriction enzymes, and the fragment of Nta-miR6155 was inserted into pCAMBIA2300 vector by using In-Fusion Snap Assembly Master Mix kit (Takara) to generate Nta-miR6155 overexpression vector.

Primers for generation of *NtCIPK18* overexpression vectors were designed based on the CDS of the *NtCIPK18* gene by using Primer Premier 5.0 software (**Table S1**). The coding sequences of *NtCIPK18* were amplified using the corresponding primers, and *NtCIPK18* fragments were fused with the *GUS* (*β-glucuronidase*) gene. The fragments with the fused GUS gene were introduced into the entry vector P35S-8GWN. Finally, the *NtCIPK18* genes with a GUS tag were recombined into the binary vector KANA303 by using Gateway LR Clonase (Thermo Fisher Scientific) to generate overexpression vector. The detail steps of plasmid construction were performed as described elsewhere (Li et al., 2015). The plasmid was transformed into *Agrobacterium tumefaciens* for further experiments.

### 2.3 *Agrobacterium*-mediated transient expression in *N. benthamiana* leaves

*Agrobacterium* containing Nta-miR6155 or *NtCIPK18-GUS* overexpression plasmid was resuspended in an infiltration medium (10 μM MES, 10 μM MgCl_2_, 200 μM acetosyringone and 500 mg glucose), and an empty vector (EV, 2300YFP) was used as a control. After transient expression of Nta-miR6155 for 36h, the infiltrated leaves were inoculated with *P. nicotianae*. Injections were performed based on the methods of previous studies (Yu et al., 2020) and each infiltration experiment was repeated more than 3 times.

### 2.4 *P. nicotianae* inoculation experiments

*N. tabacum* seedlings at 5-6 true leaf stage and *N. benthamiana* seedlings at 7-8 true leaf stage were selected for *P. nicotianae* infection, respectively. *N.tabacum* or *N. benthamiana* leaves of similar size were inoculated with *P. nicotianae* and placed in a high temperature and humidity environment (30°C and 100% relative humidity) to promote *P. nicotianae* infection. For entire plants, the stem-base of tobacco seedling was inoculated with *P. nicotianae* and placed at 30°C and 100% relative humidity. After 48 hours post-inoculation (48 hpi), the lesion diameters were measured, and lesion size and disease index were calculated according to previous methods (Zhang et al., 2021). Three biological replicates were performed for each experiment.

### 2.5 RNA extraction, cDNA preparation, and RT-qPCR

The processing conditions for plant samples are described in the main text or figure legends in detail. After collecting plant samples, total RNA was extracted with the RNAprep Pure Plant Plus Kit (TIANGEN). RNA was reverse transcribed into cDNA by using PrimeScript™ RT reagent Kit with gDNA Eraser (TaKaRa). RT-qPCR was performed by TB Green Premix Ex Taq II (TaKaRa) and miRcute Plus miRNA qPCR Kit (TIANGEN). The following default program was used: 94°C for 5 min, followed by 40 cycles of 94°C for 15 s and 60°C for 30 s each, and a dissociation stage of 95°C for 15 s, 60°C for 30 s, and 95°C for 15 s. *GAPDH* was selected as internal reference genes to calculate the relative expression levels of mRNA. All reactions were conducted with 3 biological replicates, and the relative expression of genes was conducted using the 2^-ΔΔc(t)^ method. Primers used for qRT-PCR assay are shown in **Table S1**.

To measure miRNA expression, RNA was reverse-transcribed into cDNA using miRcute Plus miRNA First-Strand cDNA Kit (TIANGEN), and RT-qPCR was performed using miRcute Plus miRNA qPCR Kit (TIANGEN). The following default program was used: 95°C for 15 min, followed by 40 cycles of 94°C for 20 s and 60°C for 30 s each, and a dissociation stage of 95°C for 10 s, 60°C for 5 s, and 95°C for 5 s. GAPDH and *U6* were selected as internal reference genes to calculate the relative expression levels of mRNA and miRNA, respectively. All reactions were conducted with 3 biological replicates, and the relative expression of genes was calculated using the 2^-ΔΔc(t)^ method. Primers used for qRT-PCR assay are shown in **Table S1**.

To relatively quantify the biomass of *P. nicotianae* in control HD and miR6155-OE plants, 100mg stem tissue was collected at 48 hpi for genomic DNA extraction. The purified genomic DNA was used as templates for RT-qPCR to relatively quantify the biomass of *P. nicotianae*. The RT-qPCR program is consistent with the program used for detecting the expression of mRNA. All reactions were conducted with 3 biological replicates, and the relative expression of genes was conducted using the 2^-ΔΔc(t)^ method. Primers used for qRT-PCR assay are shown in **Table S1**.

### 2.6 3,3’-diaminobenzidine, Nitro Blue Tetrazolium and β-glucuronidase staining and salicylic acid measurement in miR6155-OE and control HD plants post *P. nicotianae* infection

48 h post *P. nicotianae* infection, the leaves of control HD and miR6155-OE plants were collected for NBT and DAB staining, according to previously described methods (Chen et al., 2015). The GUS staining was performed to identify the expression of *NtCIPK18* according to methods described previously (An et al., 2022). Extraction of SA from tobacco and the quantification of the total SA content in plant were performed using Plant SA ELISA test kit (MEIMIAN) following the manufacturer’s instructions.

### 2.7 Phenotypic observation and nitrogen quantification in miR6155-OE and control HD plants under normal conditions

Various phenotypic characteristics including plant height, internode length, and fresh weight were measured in miR6155-OE and control HD tobacco seedlings at 5-6 true leaf stage. Subsequently, photosynthetic rate was measured in leaves of similar size using a portable photosynthetic system LI-6400. Further the nitrogen content was quantified in the seedlings using the Kjeldahl method. To analyze antioxidant enzymes and phenylalanine ammonia lyase activities in miR6155-OE and HD tobacco seedlings, After miR6155-OE and HD tobacco seedlings reached 5-6 true leaves stages, the leaves of miR6155-OE plants and HD were inoculated with *P. nicotianae* for 48h, and enzymes activities were detected by using Peroxidase (POD)Assay Kit (Solarbio), Superoxide Dismutase(SOD) Assay Kit (Solarbio), Catalase (CAT) Assay Kit (Solarbio), Total Antioxidant Capacity (T-AOC) Assay Kit (Solarbio) and Phenylalnine Ammonialyase (PAL)Assay Kit (Solarbio) in accordance with the manufacturer’s instructions.

### 2.8 Statistical analysis

Statistical analysis of data was performed with the software SPSS 22.0 (United States). Student’s t test was used to determine the significance of differences (*P<0.05; **P<0.01).

## 3 Results

### 3.1 Nta-miR6155 modulates response towards *P. nicotianae* infections in tobacco plants

**Figure 1:**
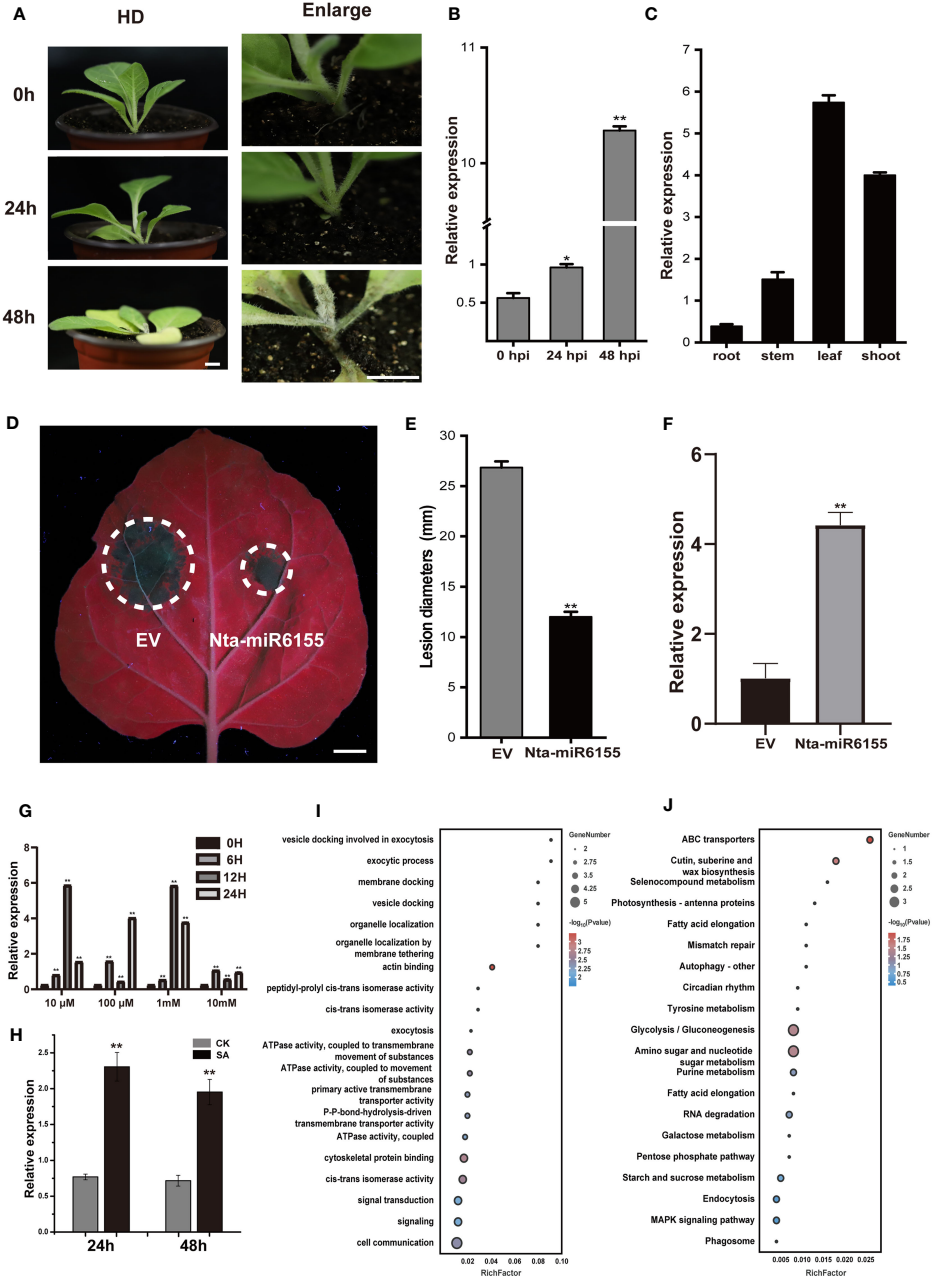
Transient expression of Nta-miR6155 in *N. benthamiana* enhanced resistance towards *P. nicotianae*. **(A)** The phenotype of tobacco after inoculation with *P. nicotianae*, bar=1cm. **(B)** The expression level of Nta-miR6155 in tobacco leaves on infected with *P. nicotianae* at 0, 24, and 48 hpi (hours post infection). **(C)** The expression level of Nta-miR6155 in different tobacco tissues, including root, stem, leaf, and shoot. **(D)** Representative photographs of *N. benthamiana* leaves after 48h infection. *Agrobacterium*-mediated transient expression of empty vector (EV, left) and Nta-miR6155 (right), bar=1cm. The infiltrated leaves (36 hours after infiltration) were inoculated with *P. nicotianae*, and photographed after 48 hpi. **(E)** Lesion diameters (mm) of *N. benthamiana* leaves. **(F)** The expression level of Nta-miR6155 in *N. benthamiana* leaves after transient expression of EV (left) and Nta-miR6155 (right). **(G, H)** The expression level of Nta-miR6155 in tobacco after treatment with salicylic acid (SA). HD seedlings at 5-6 true leaf stage were sprayed with SA at different concentrations (10 μM, 100 μM, 1mM, and 10mM), and double distilled water (ddH_2_O) was used as control (CK). After 3h, 6h, 12h, and 24h, the seedlings were collected for RNA extraction **(G)**. HD seedlings at 5-6 true leaf stage were sprayed with SA at concentration 1mM, and ddH_2_O was used as control (CK). After 24h and 48h, the seedlings were collected for RNA extraction **(H)**. **(I)** GO enrichment based on prediction of Nta-miR6155 target genes. **(J)** KEGG enrichment based on prediction of Nta-miR6155 target genes. The data was calculated from three independent experiments and statistically analyzed by Student’s t test (*P<0.05, **P < 0.01).

As a highly pathogenic oomycete, *P. nicotianae* can quickly infect tobacco plants within 48 hours under suitable conditions (**Figure 1A**). During this process, we found that the expression of Nta-miR6155, a miRNA specific to *Solanaceae* crops, is significantly induced by *P. nicotianae* infection in *N.tabacum* (**Figure 1B**; **Figure S1**) (Tang et al., 2012). Consistently, the ortholog of Nta-miR6155 in *N.benthamiana* is also induced by *P. nicotianae* infection (**Figure S2**).The tissue expression pattern of Nta-miR6155 showed that it was differentially expressed in root, stem, leaf, and shoot, suggesting that Nta-miR6155 may function in different tissues of tobacco (**Figure 1C**). To determine whether it has a crucial role in conferring resistance against *P. nicotianae* infections in *Nicotiana* species, an overexpression vector of Nta-miR6155 was constructed. Transient expression of Nta-miR6155 was performed in *N. benthamiana*, and then leaves expressing Nta-miR6155 or empty vector (control) were inoculated with *P. nicotianae* to observe its infection in *N.benthamiana* by *P. nicotianae*. The results showed that the lesion size of Nta-miR6155 overexpressed group was reduced to half that of the control group, suggesting that Nta-miRNA6155 could significantly help prevent against *P. nicotianae* infection in *N.benthamiana* (**Figures 1D–F**). Salicylic acid (SA) plays important roles in plant response to biotic and abiotic stresses (Ku et al., 2018). We found that the expression of Nta-miRNA6155 can be significantly induced by salicylic acid in a dose-dependent manner, suggesting that Nta-miR6155 may be involved in salicylic acid signaling mediated regulation of abiotic stress responses (**Figures 1G, H**). Based on the mature sequence of Nta-miRNA6155, we predicted target genes of Nta-miR6155, and a total of 106 potential target genes of Nta-miR6155 using TargetFinder software (**Tables S2**, **S3**) (Bo and Wang, 2005). GO and KEGG enrichment analysis showed that potential target genes of Nta-miRNA6155 are involved in multiple biological processes, metabolic pathways, and signaling transduction, such as autophagy, MAPK signaling, photosynthesis, and wax biosynthesis, etc., suggesting that Nta-miRNA6155 may affect tobacco disease resistance by regulating these corresponding target genes (**Figures 1I, J** and **Table S3**).

### 3.2 Overexpression of miR6155 enhanced resistance against *P. nicotianae* in tobacco

**Figure 2:**
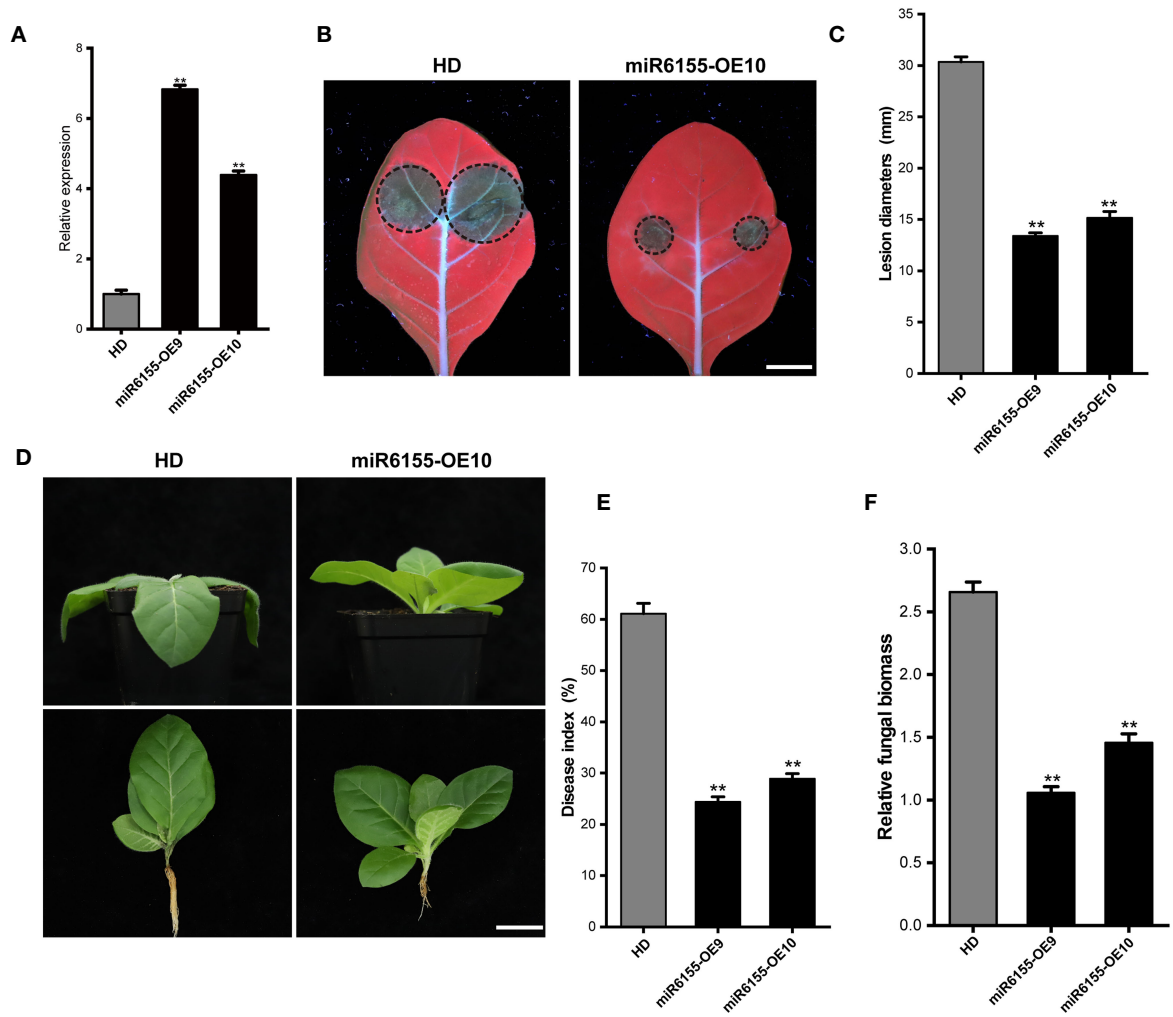
The miR6155-OE plants are resistant to *P. nicotianae*. **(A)** The expression level of Nta-miR6155 in miR6155-OE plants. At 5-6 true leaf stage, the leaves of Nta-miR6155 and control HD plants were collected to detect the expression level of Nta-miR6155 in miR6155-OE plants. **(B)** Disease symptoms in detached leaves from control HD and miR6155-OE plants at 48 hpi, bar=1.5cm. **(C)** Lesion diameters in the detached leaves from miR6155-OE plants at 48hpi with *P. nicotianae*. **(D)** Disease symptoms in control HD and miR6155-OE plants tobacco at 48hpi with *P. nicotianae*. Scale bars=3 cm. **(E, F)** Disease index and the fungal biomass in the control HD and miR6155-OE plants at 48h after inoculation. The data was calculated from three independent experiments and statistically analyzed by Student’s t test (**P < 0.01).

To further investigate the function of Nta-miRNA6155 in the tobacco plants in response to *P. nicotianae*, we generated Nta-miRNA6155 overexpression plants (miR6155-OE9 and miR6155-OE10), and RT-qPCR data showed that the expression level of Nta-miR6155 was four times higher in miR6155-OE9 and miR6155-OE10 than that of in the control HD plants (**Figure 2A**). Consistent with results in *N.benthamiana*, lesion size of detached miR6155-OE9 and miR6155-OE10 leaves were significantly smaller than those of the control HD plant leaves after inoculating with *P. nicotianae* (**Figures 2B, C**). In addition, the stem-base of miR6155-OE and HD plants were inoculated with *P. nicotianae* to observe the phenotype of miR6155-OE and HD plants after infection, and results showed that HD had been infected by *P. nicotianae* and displayed wilting and black shank phenotype. On the contrary, no obvious disease phenotype was observed in miR6155-OE9 and miR6155-OE10 under the same conditions, and the disease index and fungal biomass of miR6155-OE plants is significantly lower than that of HD (**Figures 2D–F**). These data suggest that Nta-miRNA6155 can enhance resistance to *P. nicotianae* in tobacco.

**Figure 3:**
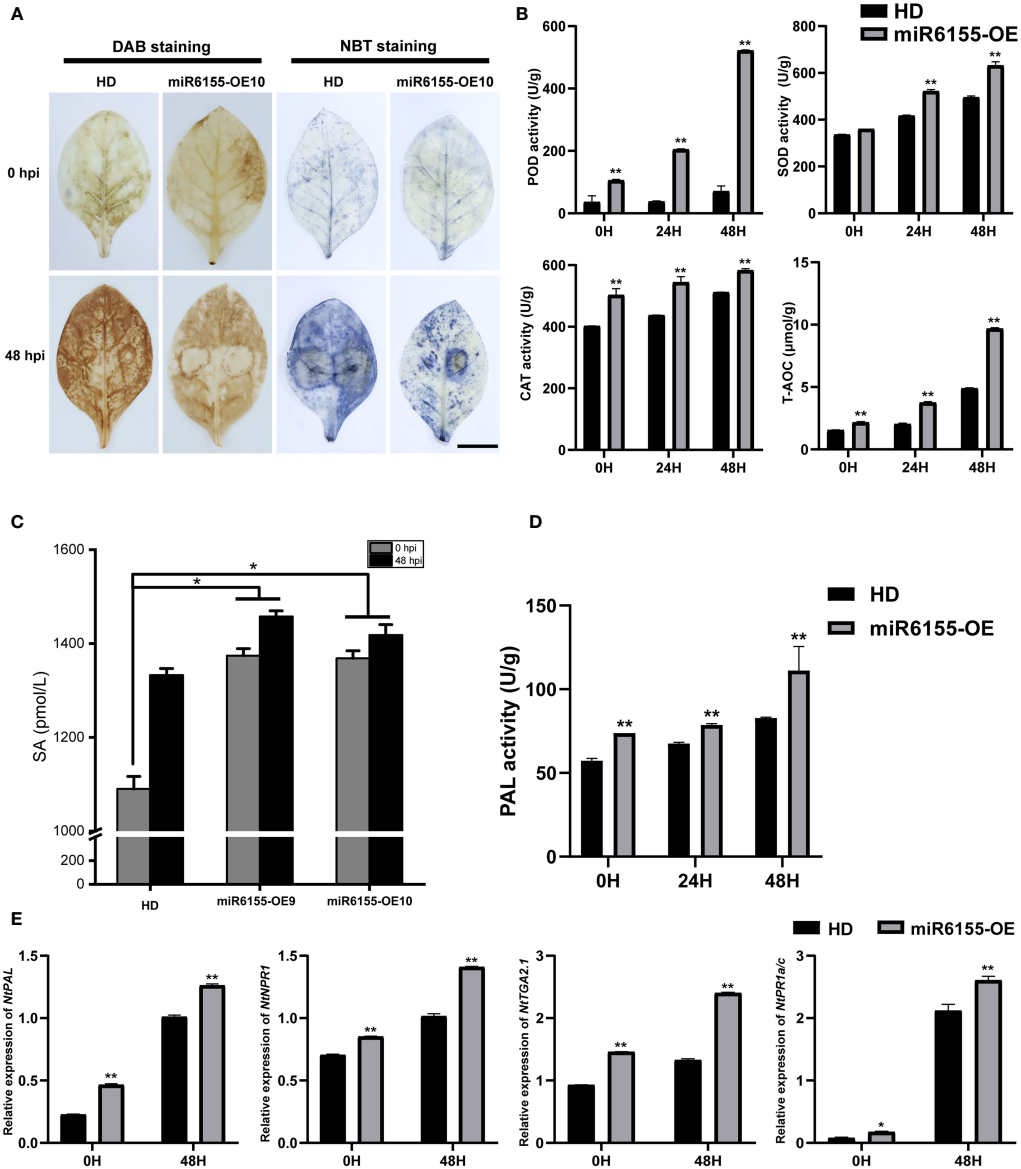
Nta-miR6155 enhances tobacco antioxidant capacities and biosynthesis and signal transduction of salicylic acid. **(A)** DAB and NBT staining of control HD and miR6155-OE tobacco leaves at 48h post *P. nicotianae* inoculation, bar =2.5cm. **(B)** Detection of antioxidant enzyme activities in control HD and miR6155-OE plants. **(C)** SA content determination. **(D)** The PAL activities in miR6155-OE and HD plants. **(E)** The expression level of salicylic acid biosynthesis and signal transduction-related genes in tobacco leaves at 0 hpi and 48 hpi with *P. nicotianae*. The data was calculated from three independent experiments and statistically analyzed by Student’s t test (*P<0.05, **P < 0.01).

ROS as a signaling molecule plays an indispensable role in the process of disease resistance in plants. ROS accumulates rapidly when plants sense the presence of pathogens, and this process is an important signal for the activation of the plant immune system (Bi et al., 2022). We investigated whether Nta-miR6155 affected ROS accumulation in tobacco after inoculation with *P. nicotianae*. The accumulation of H_2_O_2_ and O^2-^ in tobacco leaves was examined using DAB and NBT staining methods, respectively. Under normal conditions, minimal H_2_O_2_ and O^2-^ were detected in control HD and miR6155-OE tobacco leaves. After inoculation with *P. nicotianae* for 48h, DAB and NBT staining results showed that there were higher levels of H_2_O_2_ and O^2-^ accumulation in HD compared to miR6155-OE plants (**Figure 3A**). We also tested the activity of different antioxidant enzymes in miR6155-OE plants and HD, including peroxidase (POD), surperoxide dismutase (SOD), catalase (CAT), and total antioxidant capacity (T-AOC). Consistently, we found that the activity of antioxidant enzymes is higher in miR6155-OE plants than that of in HD under normal conditions or after *P. nicotianae* inoculation (**Figure 3B**). In summary, miR6155-OE plants had stronger antioxidant activity than that of HD, and the level of ROS produced by miR6155-OE plants after *P. nicotianae* infection was significantly lower than that of HD. The higher accumulation of ROS in HD leaves after *P. nicotianae* inoculation implies that HD are subjected to more severe oxidative stress, thus resulting in reduced resistance to *P. nicotianae*.

As salicylic acid has been implicated in disease resistance in plants and the expression of Nta-miRNA6155 can be induced by SA, we also examined the concentration of SA in HD and miR6155-OE plants. The results indicated that overexpression of Nta-miR6155 induced more SA accumulation in the transgenic plants than that of HD under normal conditions or after *P. nicotianae* inoculation (**Figure 3C**). In addition, we detected the expression and enzyme activity of *NtPAL* (*Phenylalanine Ammonia Lyase*), a salicylic acid biosynthesis related gene, and data showed that the expression and enzyme activity of *PAL* is higher in miR6155-OE plants than that of in HD under normal condition or after *P. nicotianae* inoculation (**Figures 3D, E**). Furthermore, we also detected the expression of salicylic acid signal transduction related genes, including *NtNPR1* (*nonexpressor of pathogenesis-related genes 1*), *NtTGA2.1* (*TGACG sequence-specific binding protein*) and *NtPR1a/c* (*pathogenesis-related genes 1*) (Peng et al., 2021). Data showed that the expression of *NtNPR1, NtTGA2.1 and NtPR1a/c* is higher in miR6155-OE plants than that of HD under normal conditions or after *P. nicotianae* inoculation (**Figure 3D**). Consistently, we also found that the resistance of *N.benthamiana* to *P. nicotianae* was increased after spraying salicylic acid (**Figure S3**). These data indicate that Nta-miR6155 could enhance tobacco resistance to *P. nicotianae* by modulating the salicylic acid biosynthesis and signal transduction.

### 3.3 Nta-miR6155 - *NtCIPK18* modulate tobacco resistance to *P. nicotianae*

**Figure 4:**
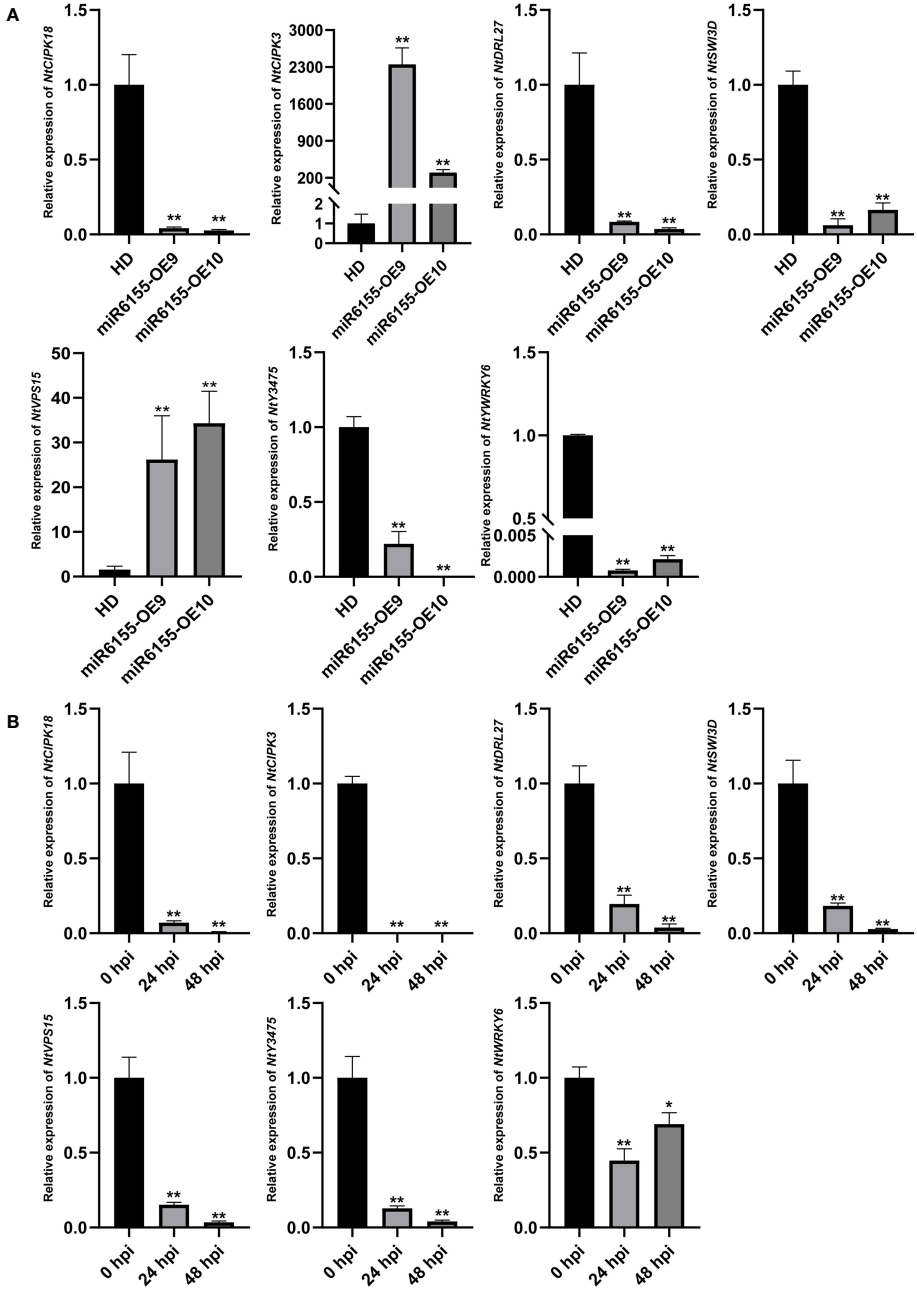
Validation of the target genes by RT-qPCR. **(A)** Expression level of target genes in miR6155-OE plants. **(B)** Expression level of target genes in tobacco leaves infected by *P. nicotianae* at 0 hpi, 24 hpi, and 48 hpi. The data was calculated from three independent experiments and statistically analyzed by Student’s t test (*P<0.05, **P < 0.01).

To shed light on the biological function of Nta-miR6155, it is of great importance to identify its direct target genes. Among the putative target genes, seven target genes were selected for RT-qPCR analysis. The results showed that most of these genes were significantly down-regulated in miR6155-OE plants compared with HD except *NtCIPK3* and *NtSVP15*, implying that Nta-miR6155 did not directly regulate *NtCIPK3* and *NtSVP15* (**Figure 4A**). In addition, the expression profile of these genes under the condition of *P. nicotianae* infection was examined. Data showed that they were significantly down-regulated after *P. nicotianae* infection (**Figure 4B**), suggesting that these genes may be involved in modulating the response to *P. nicotianae* infection in tobacco, and Nta-miR6155 may be able to regulate plant resistance through these potential target genes.

**Figure 5:**
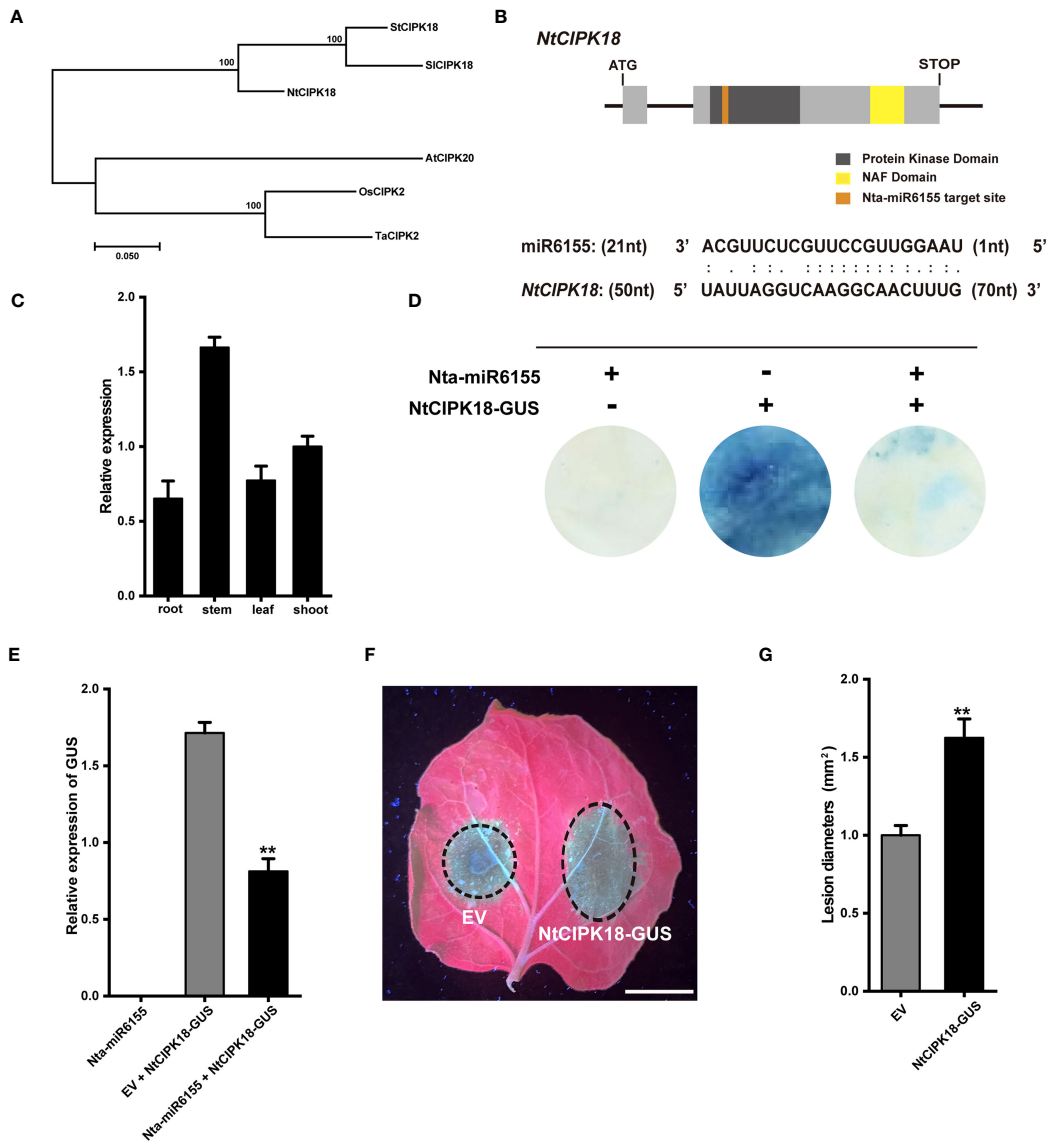
Nta-MiR6155 targets *NtCIPK18* to regulate resistance against *P. nicotianae* in *N. benthamiana*. **(A)** Phylogenetic analysis of NtCIPK18 by using MEGA 11 software shows that it is closely related to AtCIPK20 and OsCIPK2. **(B)** Diagram of *NtCIPK18* structure and the miR6155 target site (yellow box means kinase domain, brick red box means NAF domain, and green box means miR6155 target site.). **(C)** The expression pattern of *NtCIPK18* in tobacco seedling. **(D, E)** GUS staining and qRT-PCR analysis shows that miR6155 cleaves *NtCIPK18*. *P35S:: NtCIPK18-GUS* was co-expressed with P35S::*miR6155* or not, and GUS staining and qRT-PCR analysis was performed 48hpi. **(F)** Representative photographs of *N. benthamiana* leaves after 48h infection, bar=1cm. Agrobacterium-mediated transient expression of EV (left) and *P35S::CIPK18-GUS.* (right). The infiltrated leaves (after 36 h) were inoculated with *P. nicotianae*, and photographed at 48 hpi. **(G)** Lesion diameters (mm) of *N. benthamiana* leaves. The data was calculated from three independent biological replicates (**P < 0.01).

Furthermore, *NtCIPK18* was selected to further examine its role in *P. nicotianae* infections. Phylogenetic analysis showed that NtCIPK18 is closely related to SlCIPK18 and StCIPK18, and analysis of the protein domain of NtCIPK18 showed that NtCIPK18 had a conserved kinase domain and NAF domain unique to CIPK family (**Figures 5A, B**) (Tang et al., 2020). Gene expression analysis showed that the expression pattern of *NtCIPK18* was inconsistent with Nta-miR6155, and *NtCIPK18* has the highest expression in tobacco stem (**Figure 5C**). Nta-miR6155 was predicted to target *NtCIPK18* at its first exon region (**Figure 5B**), and GUS-staining and RT-qPCR analysis confirmed that *NtCIPK18* is a target of Nta-miR6155 in tobacco (**Figures 5D, E**). Finally, transient expression assay showed that overexpression of *NtCIPK18* in *N. benthamiana* reduced its resistance to *P. nicotianae*, indicating that Nta-miR6155 could partially modulate resistance to *P. nicotianae* by suppressing the function of *NtCIPK18* in tobacco plants (**Figures 5F, G**).

### 3.4 Overexpression of Nta-miR6155 delayed the growth of tobacco

**Figure 6:**
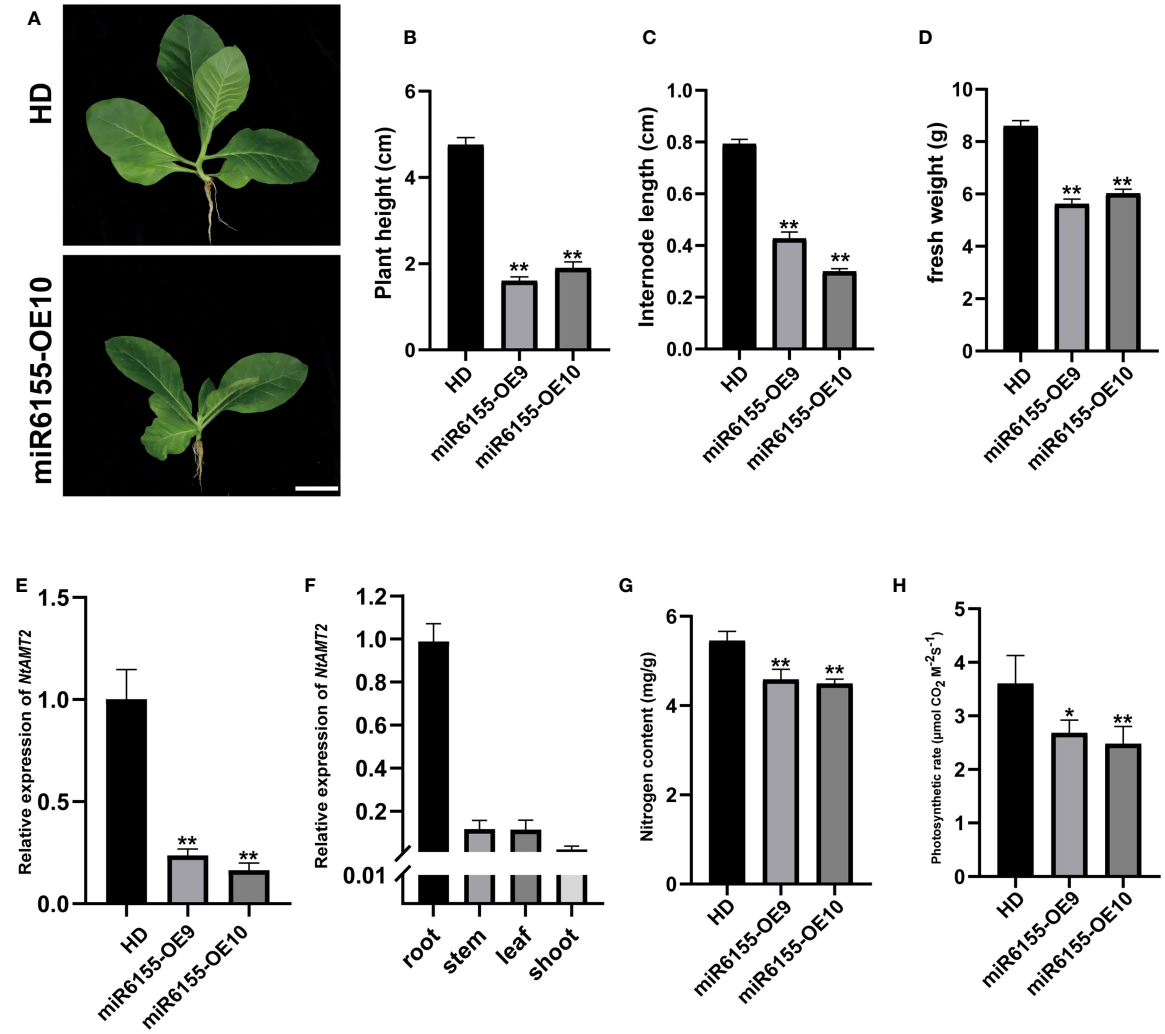
Phenotypic characterization of miR6155 transgenic plants. **(A)** The phenotype of miR6155-OE and control HD seedlings under normal conditions, bars=5 cm. **(B–D)** Plant height, internode length and fresh weight of miR6155-OE and control HD plants. **(E)** Expression level of *NtAMT2* in miR6155-OE and control HD seedlings. **(F)** The expression level of *NtAMT2* in different tobacco tissues, including root, stem, leaf and shoot. **(G, H)** Nitrogen content and photosynthetic rate in miR6155-OE and control HD plants. The data was calculated from three independent biological replicates (*P<0.05, **P < 0.01).

In addition to significant enhancement in disease resistance in miR6155-OE plants, we also found that overexpression of Nta-miR6155 suppressed growth and development of tobacco (**Figure 6A**). The plant height, internode length and fresh weight of miR6155-OE plants were significantly lower than that of the control HD plants (**Figures 6B–D**). Analyzing the target genes of Nta-miR6155, we found that *Ammonium Transport 2* (*NtAMT2*) is a potential target gene of Nta-miR6155, and *NtAMT2* encodes a high-affinity ammonium transporter. In addition, RT-qPCR data showed that the expression of *NtAMT2* was reduced fourfold in miR6155-OE plants compared with HD, and the tissue-level expression pattern analysis showed that *NtAMT2* was highly expressed in the roots (**Figures 6E, F**), implying that NtAMT2 may be involved in the absorption of nitrogen by roots. We speculate whether overexpression of Nta-miR6155 resulted in suppression of *AMT2* expression, thereby inhibiting the uptake of ammonium and growth of the miR6155-OE plants. Thus, the nitrogen content and photosynthetic rate of miR6155-OE plants and HD were measured. Consistent with our speculation, nitrogen content and photosynthetic value in miR6155-OE plants were significantly lower than that of HD (**Figures 6G, H**). These data indicate that Nta-miR6155 also plays an important role in regulating nitrogen uptake and growth and development in tobacco.

## 4 Discussion

Many studies have reported that miRNAs play essential role in plant response to biotic stresses (Robert-Seilaniantz et al., 2011; Šečić et al., 2021).For example, miR393 could regulate different target genes to modulate biotic stress response in different plants. In *Arabidopsis*, pattern-triggered immunity could induce the expression of Ath-miR393, which further represses the expression of ARF1/9 to modulate *Arabidopsis* response to biotrophic and necrotrophic pathogens by increasing glucosinolate and decreasing camalexin levels (Robert-Seilaniantz et al., 2011). In soybean, miR393 is induced by PAMPs and act as a positive regulator in soybean response to *Phytophthora sojae* (Wong et al., 2014). Besides miR393, there are many other miRNAs involved in plant response to various biotic stress in plants, such as miR160, miR167, miR164, miRNA166 and miR398, are also known to be the regulators of plant biotic stress response (Šečić et al., 2021). The expression of miR160 could be affected by different pathogens, and like miR393, miR160 can target ARFs to modulate plant response to MAMP responses in plants (Rhoades et al., 2002; Li et al., 2010). MiR164 can target NAC transcription factors to regulate auxin homeostasis and plant cell death responses in plants (Guo et al., 2005; Lee et al., 2017). miR398 could negatively regulate callose deposition and PTI by targeting superoxide dismutases (*SODs*) in *Arabidopsis* (Li et al., 2010). Recent studies have shown that miRNAs play an important role in response to *P. nicotianae* infections in tobacco plants. The overexpression of tomato miR396a-5p in tobacco has been shown to increase its susceptibility to *P. nicotianae* (Chen et al., 2015), and silencing of miR159 could enhance tobacco resistance to *P. nicotianae* infections (Zheng et al., 2020). In *Arabidopsis*, miR398b could negatively regulate plant immunity to *P. parasitica* by suppressing the expression of the *core-2/I-branching beta-1,6-N-acetylglucosaminyltransferase* gene (*AtC2GnT*) (Gou et al., 2022). However, so far, only a few miRNAs have been found to be associated with *P. nicotianae*, and studies were focused on the conserved miRNA family in plants. Research on species-specific miRNAs is limited. In this study, we found that miRNA6155, a species-specific miRNA in the *Solanaceae* species, can significantly enhance resistance to *P. nicotianae* in tobacco. Recent miRNA omics also identified many species-specific miRNA in different species (Islam et al., 2015; Chhapekar et al., 2021; Lopez-Ortiz et al., 2021). The predicted target genes of miRNAs indicate that these miRNAs can target different functional genes, including protein kinases, resistance genes and transcription factors (Chhapekar et al., 2021). In previous studies, it has found that some *Solanaceae* specific miRNA could regulate innate immunity by targeting *R* genes and *NLR* (*nucleotide-binding leucine-rich repeat*) genes (Li et al., 2012; Deng et al., 2018). Further research will help reveal the role of species-specific miRNA in plant development and defense signaling pathways.

*P. nicotianae* causes significant economic losses in tobacco cultivation, and only a few genes that can participate in tobacco response to *P. nicotianae* have been identified in previous studies, such as *NpPP2-B10* and *EIN3* (Cho et al., 2013; Wen et al., 2023). MiRNA mainly regulates the function of target genes at the post transcriptional level by directly cleaving target genes or inhibiting their translation. In this study, we predicted target genes of Nta-miR6155, and RT-qPCR data showed some target genes were significantly down-regulated in Nta-miR6155 overexpression lines. Nta-miR6155 may affect the tobacco resistance to *P. nicotianae* by modulating the function of its target genes. CIPKs belong to a subclass of serine/threonine (Ser/Thr) protein kinases, and play crucial roles in plants development and stress response (Yang et al., 2022). In previous studies, it has been found that CIPKs were involved in plant response to biotic stress by regulation of reactive oxygen species (ROS) production (Yang et al., 2022). For instance, CIPK26 and CIPK6 could promote ROS production through NADPH oxidases (de la Torre et al., 2013; Kimura et al., 2013). TaCIPK10 could interact and phosphorylate TaNH2, a homologous of AtNPR3/4, and phosphorylate it to enhance wheat resistance to wheat stripe rust (Liu et al., 2019). In this study, we showed that *NtCIPK18* was significantly suppressed under *P. nicotianae*, and transient expression of *NtCIPK18* resulted in significantly reducing *N. benthamiana* resistance to *P. nicotianae*, suggesting that *NtCIPK18* is a negative regulator of plant resistance to *P. nicotianae*.

Furthermore, our data showed that overexpression of miR6155 resulted in slow growth and smaller internode length in tobacco. In previous studies, miRNAs have been found to be involved in plant growth and development under nitrogen starvation conditions (Liang et al., 2012; Kant, 2018). He et al. showed that miR826 and miR5090 are involved in mediating nitrogen starvation adaptation by regulating *Alkenyl Hydroxalkyl Producing2* (*AOP2*) (He et al., 2014). Overexpression of miR169a resulted in significantly decreased nitrogen starvation tolerance in *Arabidopsis* (Zhao et al., 2011). Interestingly, we found that *AMT2*, encoding a high-affinity ammonium transporter, is a potential target gene of miR6155. In previous studies, it has been shown that AMT2 potentially regulates ammonium uptake in different plants (Zhang et al., 2022). Additionally, Giehl et al. has shown that *AMT2;1* was crucial for root-to-shoot translocation of ammonium in *Arabidopsis* (Giehl et al., 2017). Our RT-qPCR data showed that the expression level of *AMT2* was significantly down-regulated in miR6155-OE plants, and nitrogen content also significantly decreased in miR6155-OE plants, suggesting that miR6155 may participate in regulating ammonium uptake through *AMT2*. These results indicate that miRNA6155 plays an important role not only in regulating disease resistance but also in nutrient absorption.

Overall, we identified and investigated the function of miR6155 in tobacco in response to *P. nicotianae* infection. Meanwhile, our results indicate that species-specific miRNAs have important functions in regulating growth and development and stress response in plants, and future research on these species-specific miRNAs may provide more important genetic resources for stress-resistance targeted crop breeding.

## Data availability statement

The original contributions presented in the study are included in the article/**Supplementary Material**. Further inquiries can be directed to the corresponding authors.

## Author contributions

KY: Data curation, Formal Analysis, Writing – original draft. YH: Formal Analysis, Methodology, Writing – original draft. ZL: Data curation, Formal Analysis, Writing – review & editing. QZ: Data curation, Formal Analysis, Writing – original draft. XD: Formal Analysis, Writing – review & editing. JL: Formal Analysis, Writing – original draft. XZ: Formal Analysis, Writing – original draft. KD: Conceptualization, Methodology, Writing – original draft, Writing – review & editing. JZ: Conceptualization, Methodology, Writing – original draft, Writing – review & editing.

## Supplementary material

Supplementary Figure 1 The expression level of Nta-miR6155 at early time points after *P. nicotianae* inoculation.

Supplementary Figure 2 The ortholog of Nta-miR6155 in *N.benthamiana* is induced by *P. nicotianae*. **(A)** The phenotype of *N.benthamiana* after inoculation with *P. nicotianae*, bar=2 cm. **(B)** The expression level of miR6155 in *N.benthamiana* on infection with *P. nicotianae* at 48 hpi.

Supplementary Figure 3 Salicylic acid enhances resistance against *P. nicotianae* in *N.benthamiana*. bar=1 cm. **(A)** Representative photographs of *N. benthamiana* leaves after 48h infection. bar= 1 cm. *N. benthamiana* seedlings at 7-8 true leaf stage were sprayed with SA (1mM) and double distilled water (ddH2O) was used as control (CK). After 24h, the leaves of *N. benthamiana* seedlings were inoculated with *P. nicotianae*, and photographed at 48 hpi. **(B)** Lesion diameters (mm) of *N. benthamiana* leaves.
