# Supplementary figures and images for: Overexpression of Nta-miR6155 confers resistance to Phytophthora nicotianae and regulates growth in tobacco (Nicotiana tabacum L.)
Source: Front Plant Sci. 2023 Nov 20;14:1281373. doi: 10.3389/fpls.2023.1281373 (PMC10694243; doi:10.3389/fpls.2023.1281373)

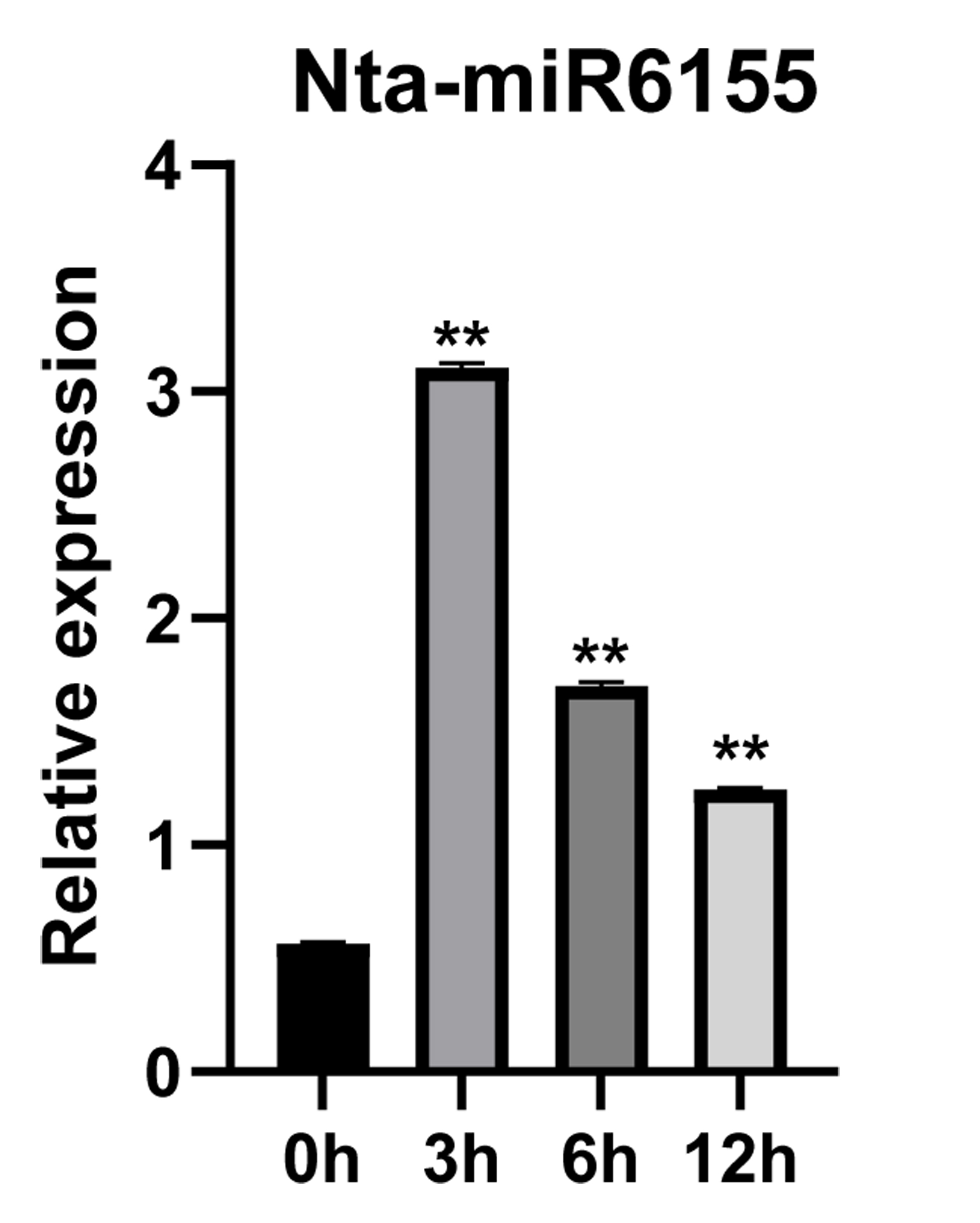

Supplement: Supplementary Figure 1 — The expression level of Nta-miR6155 at early time points after P. nicotianae inoculation. [file Image_1.jpeg]

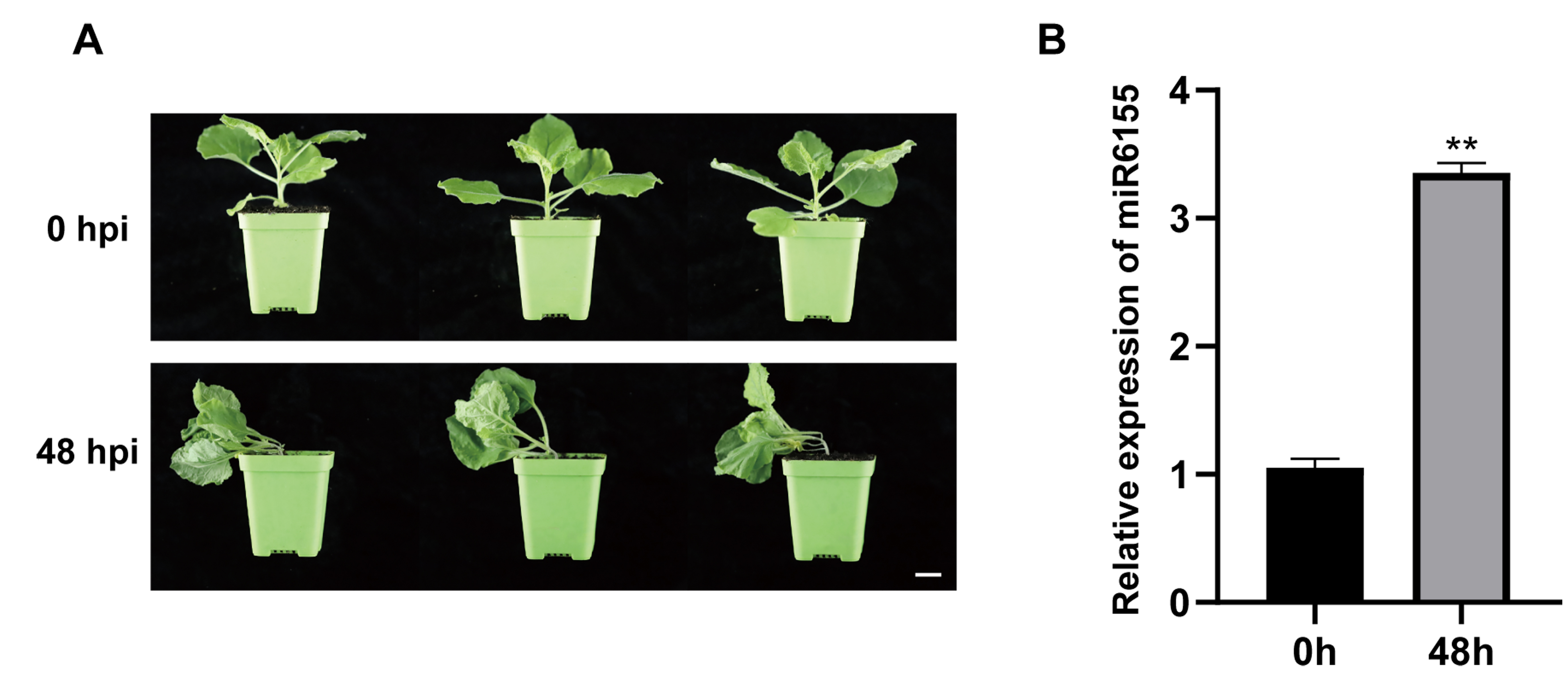

Supplement: Supplementary Figure 2 — The ortholog of Nta-miR6155 in N.benthamiana is induced by P. nicotianae. (A) The phenotype of N.benthamiana after inoculation with P. nicotianae, bar=2 cm. (B) The expression level of miR6155 in N.benthamiana on infection with P. nicotianae at 48 hpi. [file Image_2.jpeg]

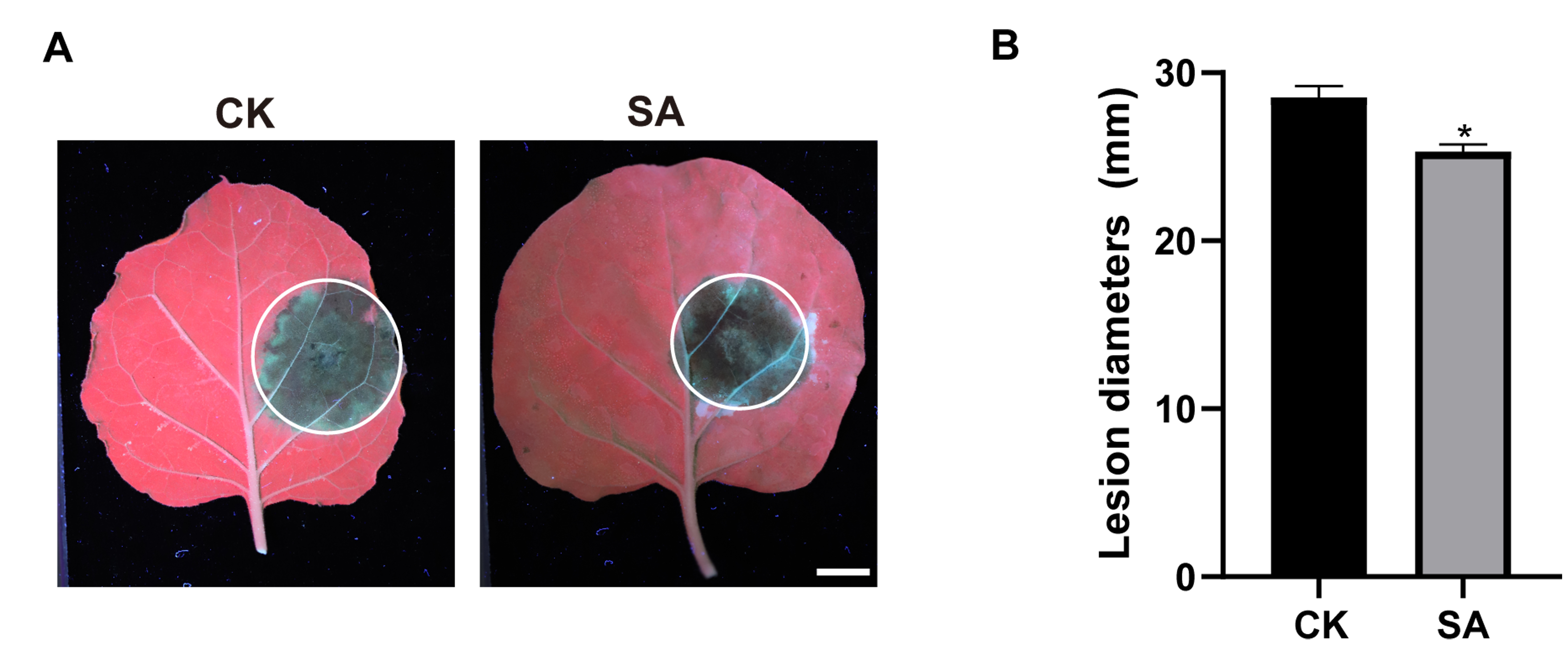

Supplement: Supplementary Figure 3 — Salicylic acid enhances resistance against P. nicotianae in N.benthamiana. bar=1 cm. (A) Representative photographs of N. benthamiana leaves after 48h infection. bar= 1 cm. N. benthamiana seedlings at 7-8 true leaf stage were sprayed with SA (1mM) and double distilled water (ddH2O) was used as control (CK). After 24h, the leaves of N. benthamiana seedlings were inoculated with P. nicotianae, and photographed at 48 hpi. (B) Lesion diameters (mm) of N. benthamiana leaves. [file Image_3.jpeg]
